# Plastid RNA Editing in *Glycyrrhiza uralensis*: Landscape Characterization and Comparative Assessment of RNA-Seq Library Strategies for Detection

**DOI:** 10.3390/genes16101142

**Published:** 2025-09-26

**Authors:** Hui Ma, Yixuan Rao, Yinxiao Lu, Na Fang, Yijia Huang, Lei Gong

**Affiliations:** 1 College of Agriculture and Bioengineering, Longdong University, Qingyang 745000, China; 19393490921@163.com (Y.R.); 19119733081@163.com (Y.L.); 18794482597@163.com (N.F.); 13647054803@163.com (Y.H.); gl888168@163.com (L.G.); 2 Gansu Key Laboratory of Conservation and Utilization of Biological Resources and Ecological Restoration in Longdong, Qingyang 745000, China

**Keywords:** plastid RNA editing, library strategy, *Glycyrrhiza uralensis*

## Abstract

Background: Plastid RNA editing is widespread in angiosperms yet remains underexplored in the medicinal non-model species *Glycyrrhiza uralensis*. This study aimed to (i) comprehensively identify plastid RNA editing sites in *G. uralensis*, and (ii) compare the detection performance of three library construction strategies: total RNA-seq, rRNA-depleted RNA-seq, and mRNA-seq. Methods: Leaf tissue was used from three wild-sampled individual plants. Plastomes were assembled with GetOrganelle v1.7.0 and annotated using PGA. Strand-specific RNA-seq libraries were mapped to sample-matched plastomes using HISAT2 v2.2.1. Variants were identified using REDItools v2.0 under uniform thresholds. Candidate sites were visually verified in IGV v2.12.3, and read origins were confirmed by BLAST v2.13.0+; artifacts were removed via strand-specific filtering. Results: After stringent filtering, 38 high-confidence RNA editing sites were identified across 19 genes. Total RNA seq performed the best, detecting 37/38 sites consistently, whereas rRNA-depleted libraries detected fewer genuine sites and produced numerous rRNA-linked, noncanonical, noncoding-strand-dominant artifacts. Despite very low rates of plastid mapping, mRNA seq recovered a large fraction of bona fide sites under stringent, strand-aware filtering. Conclusions: We establish a set of 38 high-confidence plastid RNA editing sites in *G. uralensis* and suggest potential adaptive implications of editing in *ndh*-related genes. Methodologically, total RNA-seq is recommended for identification using de novo RNA editing due to its high sensitivity and low false-positive rate; publicly available poly(A)-selected mRNA-seq datasets can be repurposed to reliably retrieve plastid RNA editing sites when stringent strand-specific filtering is applied.

## 1. Introduction

RNA editing serves as a crucial post-transcriptional mechanism that modifies specific nucleotides in RNA. In land plants, this process occurs predominantly within plastids and mitochondria, where it involves cytidine-to-uridine (C→U) conversions, and less commonly, U→C changes [1,2,3,4]. These edits often restore evolutionarily conserved codons, rectify genomic mutations, and influence RNA stability or processing, thereby playing an indispensable role in organellar gene regulation and function [5,6]. In chloroplasts, RNA editing targets key photosynthetic components, including electron transport chains and ATP synthesis machinery, directly affecting photosynthetic performance and plant stress adaptation [7].

The advent of high-throughput RNA sequencing (RNA-Seq) has revolutionized genome-wide profiling of RNA editing, enabling comparative studies across species, tissues, and environments. Accurate detection, however, demands high sequencing depth, strand specificity, and meticulous bioinformatic removal of genomic polymorphisms and technical artifacts, including sequencing errors, misalignment, and nuclear copies of organellar DNA. The library preparation strategy represents a frequently underestimated variable in editing studies. Although poly(A) selection excels in enriching polyadenylated nuclear transcripts [8], it poorly represents non-polyadenylated plastid RNAs. In contrast, rRNA depletion and total RNA protocols retain a broader spectrum of organellar transcripts, yet differ in background noise, rRNA removal efficiency, and cost, resulting in substantial variations in editing detection performance. Strand-specific methods, such as dUTP-based library construction, are particularly effective at storing strand information of transcripts [9], facilitating accurate gene expression quantification and variant calling.

While previous comparisons of library types have largely focused on nuclear transcriptomics [10,11,12,13], systematic evaluations aimed at plastid RNA editing remain limited. Most studies in model plants and crops employ either total RNA or rRNA-depleted libraries to maximize organellar coverage; however, comparative assessments of all three strategies, including poly(A)-selected, rRNA-depleted, and total RNA, using matched genomic controls and strand-specific transcriptional data are still scarce. Library selection critically influences the detection sensitivity, reproducibility, and accuracy of RNA editing analyses, highlighting the need for empirical, species-specific guidance.

Legumes are of considerable ecological and economic importance. Although plastid RNA editing has been characterized in some legume species [14,15], the impact of library construction strategies on RNA editing detection remains poorly explored in non-model legumes, especially those with medicinal value or that have adapted to stressful environments. In the study of RNA editing in *Vigna* species [14,15], two different approaches—rRNA-depleted RNA-seq and poly(A)-enriched mRNA-seq—yielded a comparable number of RNA editing sites (41 vs. 34). This result suggests the potential utility of reusing mRNA-seq data for RNA editing studies, which is a particularly valuable opportunity given the rapidly expanding volume of mRNA datasets in public databases. *G*. *uralensis*, a perennial legume, is a medicinally important species widely used in traditional medicine for its tonic properties. It thrives across northern regions of the Yellow River basin in China, including Shaanxi, Gansu, Inner Mongolia, and Qinghai, where it holds significant medicinal and ecological value.

The primary objective of this study is to deliver the first comprehensive characterization of the plastid RNA editing landscape in *G. uralensis* through a systematic comparison of three RNA-Seq library strategies: poly(A)-selected mRNA-seq, rRNA-depleted RNA-seq, and total RNA-seq. As secondary objectives, we evaluate the detection performance of these RNA-Seq library strategies in terms of sensitivity, specificity, and reproducibility, characterize antisense-derived artifacts, and assess the feasibility of re-purposing existing poly(A)-selected mRNA-seq datasets for plastid RNA editing analyses, thereby clarifying trade-offs among library strategies and guiding experimental design in organellar RNA research.

## 2. Materials and Methods

### 2.1. Sample Collection, Nucleic Acid Extraction, Library Preparation, and Sequencing

Fresh leaves of *G. uralensis* were collected from three wild individuals in Qingyang, Gansu Province, China. Samples were rinsed briefly with deionized water, blotted dry, flash-frozen in liquid nitrogen on-site, transported on dry ice to the laboratory, and then stored at −80 °C until processing. The collection complied with local regulations, and no endangered species were sampled.

All wet-lab experiments were conducted using Anhui Double Helix Gene Technology Co., Ltd., Hefei, Anhui, China. Each sample was used for both DNA and RNA extraction. Total genomic DNA was extracted from frozen leaf tissue per sample using a modified cetyltrimethylammonium bromide (CTAB) protocol [16]. The quality of extracted DNA was evaluated using 0.8% agarose gel electrophoresis, and DNA quantity was measured with a UV spectrophotometer. Qualified DNA was used for library preparation with an library size of ~450 bp according to the standard workflow of the TruSeq DNA PCR-Free Prep Kit (Illumina, San Diego, CA, USA), following the manufacturer’s instructions. Prior to sequencing, library quality control (QC) was performed on an Agilent Bioanalyzer using the Agilent High-Sensitivity DNA Kit (Agilent Technologies, Santa Clara, CA, USA). Qualified libraries displayed a single, distinct peak without detectable adapter dimers. Library quantification was conducted using the Quant-iT PicoGreen dsDNA Assay Kit (Thermo Fisher Scientific, Waltham, MA, USA) on a Promega QuantiFluor fluorescence quantification system (Promega, Madison, WI, USA). Libraries with concentrations ≥ 2 nM were considered acceptable for sequencing. Qualified libraries (with non-redundant index sequences) were gradient-diluted, pooled at appropriate ratios according to the required sequencing depth, and denatured with NaOH to generate single-stranded libraries for loading. Paired-end sequencing (2 × 150 bp) was performed on an Illumina NovaSeq 6000 platform (Illumina, San Diego, CA, USA).

Total RNA was extracted using TRIzol Reagent (Invitrogen, Carlsbad, CA, USA) following the manufacturer’s protocol; RNA purity was assessed using spectrophotometry (NanoDrop 2000, Thermo Fisher Scientific, Waltham, MA, USA); and RNA integrity was evaluated on an Agilent 2100 Bioanalyzer with the RNA 6000 Nano kit (Agilent Technologies, Santa Clara, CA, USA).

We evaluated three strand-specific RNA-seq strategies that differed only in the initial RNA processing step; all subsequent library construction steps were identical after fragmentation and are described in the following paragraph. For total RNA-seq, total RNA (≥0.4 µg) was used directly without prior enrichment or depletion for fragmentation. For rRNA-depleted RNA-seq, rRNA was removed from ≥0.4 µg total RNA using the Epicentre Ribo-Zero rRNA Removal Kit (Plant) (Illumina/Epicentre, Madison, WI, USA) according to the manufacturer’s instructions. The depleted RNA was purified and immediately processed for fragmentation. For poly(A)-selected mRNA-seq, polyadenylated mRNA was enriched from ≥0.4 µg total RNA using oligo (dT) magnetic beads included in the NEBNext Ultra Directional RNA Library Prep Kit for Illumina (New England Biolabs, Ipswich, MA, USA). The captured mRNA was washed, eluted, and then fragmented.

After the initial processing described above, all three strategies followed the same strand-specific library preparation steps. RNA was fragmented using divalent cations at elevated temperature (kit-supplied fragmentation buffer) to achieve an average library size of ~400–500 bp. Fragmented RNA was reverse-transcribed using random hexamer primers and reverse transcriptase (e.g., SuperScript II, Thermo Fisher Scientific, Waltham, MA, USA). Second-strand synthesis was performed using DNA polymerase I and RNase H with a dNTP mix containing dUTP (replacing dTTP) to mark the second strand for strandedness assessment. Blunt ends were generated, and 3′ A overhangs were added using kit enzymes to prepare for adapter ligation. Illumina paired-end adapters with unique dual indices (UDIs) were ligated to A-tailed fragments (Illumina, San Diego, CA, USA). The USER enzyme (New England Biolabs, Ipswich, MA, USA) was applied to selectively degrade the dUTP-containing second strand, yielding reverse-forward (RF) stranded libraries. Libraries were purified and size-selected using AMPure XP beads (Beckman Coulter, Brea, CA, USA) at ~400–500 bp. Adapter-ligated fragments were amplified with limited PCR cycles (typically 8–12, per input amount), then purified again with AMPure XP beads. Library quality was assessed using the Agilent 2100 Bioanalyzer with the High-Sensitivity DNA Kit (5067-4626; Agilent Technologies Inc., Santa Clara, CA, USA). Total library concentration was detected using the Quant-iT PicoGreen dsDNA Assay Kit (P7589; Invitrogen, Carlsbad, CA, USA) on a QuantiFluor-ST fluorometer (E6090; Promega, Madison, WI, USA). Effective molarity was determined by qPCR using the StepOnePlus Real-Time PCR System (Thermo Fisher Scientific, Waltham, MA, USA). Multiple DNA libraries were normalized, pooled in equal volumes, serially diluted, quantified, and sequenced on an Illumina platform in PE150 mode with typical loading concentrations of 1.5 nM and a volume of 400 µL per lane.

### 2.2. Plastid Genome Assembly, Annotation, Alignment, and Variant Calling

Raw reads (genomic and RNA) were processed with fastp v0.23.4 [17] to remove adapters, trim bases with Phred quality lower than 15 (-q 15), and filter out reads that, prior to trimming, had more than 40% of bases with Phred quality below 15 (-u 40) or contained more than five undetermined bases (-n 5). Plastid genomes were assembled from cleaned genomic reads with GetOrganelle v1.7.0 [18]. Plastid genome annotation was performed with PGA (Plastid Genome Annotator, 2020) [19] using a published *G. uralensis* reference plastome (Accession number: MZ329070.1) and curated manually to confirm gene boundaries, intron/exon structures, and start/stop codons. tRNA boundaries were verified with tRNAscan-SE v2.0.8 [20].

Because the three plastid genomes showed few structural variations, we aligned the complete plastome sequences with MAFFT v7.505 [21]. Alignments were manually checked and confirmed in MEGA v12 [22]. The reason for aligning plastomes was to ensure a consistent coordinate system for comparing RNA editing sites across samples.

### 2.3. Identification of RNA Editing Sites

Cleaned RNA-Seq reads from each library type were mapped to the corresponding sample’s plastid genome using HISAT2 v2.2.1 [23] with parameters optimized for spliced, strand-specific mapping (–dta–RNA-strandness RF). SAMtools v1.15.1 [24] was used to sort alignment files and mark PCR duplicates.

Putative RNA editing sites were identified using REDItools 2.0 [25]. Candidate sites were then filtered using custom scripts with the following conservative criteria for reporting high-confidence editing sites: (1) sites covered by ≥3 uniquely strand-specific supporting reads; (2) variant allele fractions ≥10% in at least one RNA library.

To further reduce false positives arising from mapping or library-prep artifacts, we performed the following: (i) alignment inspection in IGV v2.12.3 [26] for a subset of candidate sites; (ii) cross-library comparison to assess whether sites were reproducibly detected across library types (mRNA, rRNA-depleted, total RNA) and samples; (iii) BLAST-based checks [27] to ensure reads supporting editing sites did not map better to nuclear or mitochondrial paralogs. The mitochondrial genome (Accession number: NC_053919.1, MZ066515.1) and nuclear genome (GCA_027886165.1) of *G. uralensis* were downloaded from NCBI. We also used an in-house Perl script to validate strand concordance at each candidate site by counting variant-supporting reads on the expected strand vs. the opposite strand; sites with >10% variant reads were manually inspected.

## 3. Results

### 3.1. Plastid Genome Assembly, Comparisons, and Annotation

With whole-genome resequencing, we obtained 253 to 352 million paired-end genomic reads for the three *G uralensis* individuals. Using GetOrganelle v1.7.0, we assembled complete circular plastid genomes for all three samples based on the cleaned genomic reads. The three plastid assemblies ranged from 127,670 to 127,716 bp in length (Table 1) and differed by 20–34 single-nucleotide variations and 11–17 short insertions/deletions, totaling 61–110 variable sites in each pairwise comparison after MAFFT v7.505 alignment. The *G. uralensis* plastid genome was annotated using PGA (2020), encoding 76 proteins, 30 tRNAs, and four rRNAs.

### 3.2. RNA-Seq Alignment

We used HISAT2 v2.2.1 to align each sample’s RNA-seq reads to its corresponding plastid genome. Mapping rates were highest for rRNA-depleted RNA-seq (33%, 23%, 39%), intermediate for total RNA-seq (25%, 20%, 29%), and very low for mRNA-seq (0.14–0.48%) from Table 2. The extremely low mapping rate in mRNA-seq is consistent with its poly(A) selection, which enriches nuclear transcripts but excludes most plastid RNAs that lack poly(A) tails. Because plastid coverage in the mRNA-seq data was shallow and incomplete, we first benchmarked plastid RNA editing using reads from the rRNA-depleted and total RNA-seq libraries before evaluating the detection performance of mRNA-seq separately.

Benefiting from strand-specific RNA-seq, we could accurately resolve sequence variations on each strand. We used REDItools v2.0 to extract strand-aware variant profiles. Applying uniform thresholds of at least three strand-specific supporting reads and a minimum variant allele fraction of 10% per strand, we detected a total of 90 single-nucleotide variants (SNVs). All candidates were visually inspected in IGV v2.12.3, and read origins were further verified by BLAST v2.13.0+ against the *G. uralensis* plastid, mitochondrial, and nuclear genomes to confirm best-hit loci. This process excluded four questionable variants caused by alignment artifacts at intron boundaries, yielding 86 RNA editing candidates.

**Figure 1:**
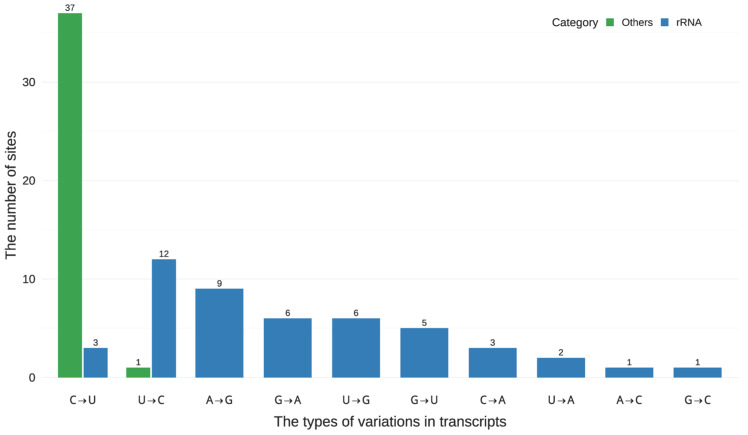
Substitution-type counts for plastid transcript variants in three *G. uralensis* leaf tissues, classified by genomic context: rRNA regions (blue) versus non-rRNA regions (green). RNA-Seq data were generated from two library strategies—rRNA-depleted RNA-seq and total RNA-seq. Variants were called using REDItools v2.0 under uniform thresholds (≥3 uniquely mapped, strand-consistent supporting reads; variant allele fraction ≥ 10%; duplicate and multimapping reads excluded); a total of 86 plastid variations were identified in these experiments. Genomic contexts were partitioned into plastid-annotated rRNA loci (“rRNA regions”) and all other loci, including protein-coding genes and intergenic sequences. Bars show counts of variant sites by substitution type. Canonical plastid edits (C→U, with occasional U→C) are concentrated in non-rRNA regions, whereas rRNA-mapped variants show diverse noncanonical changes, likely arising from reverse transcription across modified rRNA bases and/or second-strand carryover. Units: number of variant sites.

Of the 86 candidate sites included, 37 were located in protein-coding regions (including 34 at nonsynonymous or synonymous codon positions, 1 in the 5′ UTR, 1 in the 3′ UTR, and 1 within an intron), 48 were located in rRNA regions, and 1 was intergenic. Outside rRNA regions, editing events were almost exclusively C→U, with a single U→C change in the 3′ UTR. In contrast, within rRNA regions, we observed 10 distinct substitution types (Figure 1), with C→U accounting for only 6.25% of events; other substitutions, such as U→C, A→G, and G→A, were more frequent. Since canonical RNA editing in plant plastids primarily involves C→U (and occasionally U→C) transitions, the diverse substitution spectrum in rRNA regions suggests that most candidates derived from these regions are likely technical artifacts.

**Figure 2:**
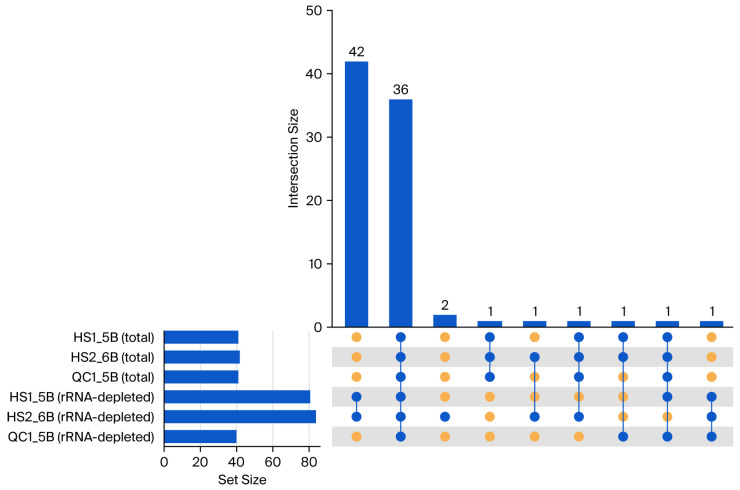
UpSet plot of chloroplast RNA editing variant-site intersections across biological replicates and library strategies in *G uralensis* leaf tissue. Six sets are the identified variant sites in three biological replicates by two library strategies (total RNA-seq and rRNA-depleted RNA-seq). Top bars indicate intersection sizes (number of sites shared by exactly the sets indicated); left bars indicate per-set sizes (number of sites in each replicate). The bottom matrix marks set membership for each intersection (blue circles = included; orange circles = excluded). This comparison highlights higher recovery and reproducibility in total RNA-seq (larger multi-replicate intersections) and a distinct artifact profile in rRNA-depleted RNA-seq (numerous singleton or low-overlap sites). Units: number of variant sites. HS1_5B, HS2_6B and QC_5B are sample IDs. Abbreviations: total, total RNA-seq; rRNA-depleted, rRNA-depleted RNA-seq.

On the sample level, total RNA-seq detected 39–44 candidate sites, while rRNA-depleted RNA-seq detected 39–83 (Figure 2). The increase observed in rRNA-depleted libraries was largely attributable to numerous candidates within rRNA regions, which exhibited low cross-library reproducibility and atypical substitution patterns. Outside rRNA genes, however, rRNA-depleted RNA-seq detected fewer candidates than total RNA-seq, indicating that rRNA depletion does not enhance detection in protein-coding regions and may introduce artifacts near rRNA loci.

Notably, 1 of the 36 universally detected sites lies within an rRNA region, showing an unconventional U→A substitution at an exceptionally high read depth, with an approximately 50% editing frequency (14,211/33,551 in HS1_5B; 12,265/25,329 in HS2_6B; 15,188/29,855 in QC1_5B for total RNA-seq). This site was, therefore, excluded. After removing all 48 rRNA-region sites, 38 RNA editing candidates remained.

Among these 38 sites, 35 were detected in all six experiments; 1 site was detected in five experiments and was present in the sixth at 9% editing frequency (just below the 10% threshold); 1 site was detected in four experiments and present in a fifth at 5% editing (absent in one rRNA-depleted library, likely due to its proximity—731 bp—to a 16S rRNA gene on the same strand); and 1 site was detected in two experiments and present in the other four at editing frequencies of 3–8%. Collectively, these 38 sites represent a high-confidence set of RNA editing candidates.

**Figure 3:**
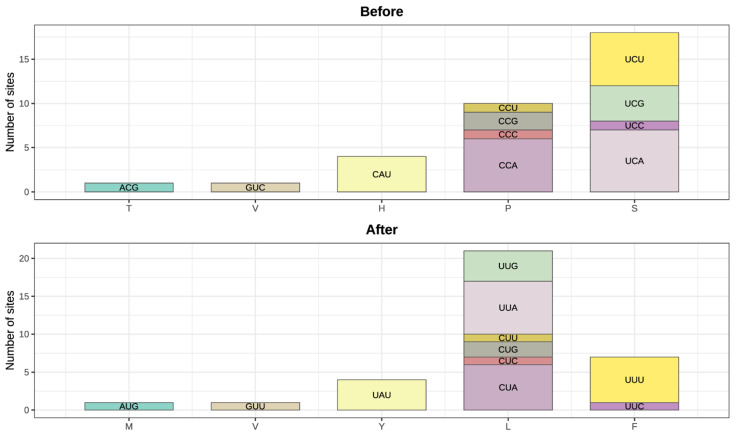
Codon and amino acid distributions for the 34 edited codons before (**top**) and after (**bottom**) RNA editing. Each stacked bar shows the number of sites per codon contributing to each amino acid class. The color of each codon corresponds to its type prior to RNA editing. Edited codons retain the same color as their unedited counterparts.

These 38 candidates are distributed across 19 protein-coding genes. *ndhB* harbors the most RNA editing sites (8), followed by *rpoB* (4) and *ndhD* (3), with the remaining genes each containing 1–2 sites (Table 3 and Table 4). At the codon level, the amino acids encoded by codons subject to RNA editing were most frequently serine (18 codons), followed by proline (10) and histidine (4) (Figure 3). After editing, leucine became the most frequently encoded amino acid (21 codons), with tyrosine and phenylalanine each encoded by 7 codons. Except for one codon (GUC), RNA editing occurred at the first and second positions of all other codons.

**Figure 4:**
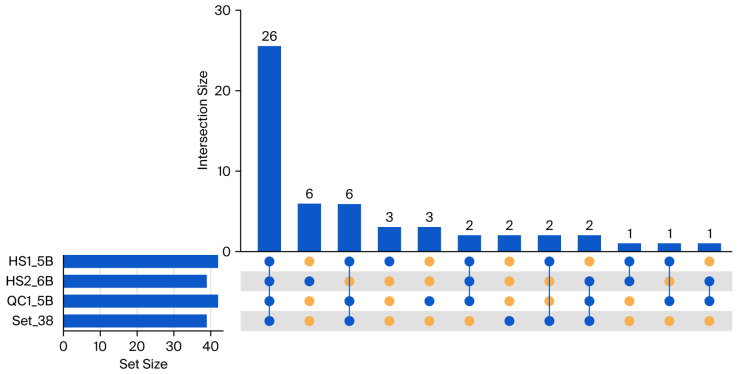
UpSet plot of plastid RNA editing variant–site intersections among three mRNA-seq samples (HS1_5B, HS2_6B, QC1_5B) and a curated reference set of 38 high-confidence plastid RNA-editing sites from *G. uralensis* leaf tissue. Sets are HS1_5B, HS2_6B, QC1_5B, and the reference set (Set_38). Top bars indicate intersection sizes (number of sites shared by exactly the indicated sets); left bars indicate per-set sizes (number of sites in each set). The bottom matrix marks set membership for each intersection (blue circles = included; orange circles = excluded). The substantial overlap between mRNA-seq-identified sites and the reference set, particularly for sites appearing in multiple mRNA-seq samples, supports the reliability of mRNA-seq for plastid RNA-editing detection. Units: number of variant sites. HS1_5B, HS2_6B and QC_5B are sample IDs.

Using the same criteria, we analyzed RNA editing in the mRNA-seq data from all three samples. Despite the low mapping rates, each mRNA-seq library yielded 38–41 candidate sites, among which 28–34 overlapped with the set of 38 high-confidence editing sites (Figure 4). In total, 28 variants were shared across all three mRNA-seq datasets, with 26 belonging to the high-confidence set. Among the 39 variants shared by any two samples, 34 variants were also included in this high-confidence set. Three out of the remaining five variants were present in total/rRNA-depleted RNA-seq at sub-threshold editing efficiency. The other two variants, as uncanonical substitutions of A→G and T→G, might result from sequencing on modified nucleotides and should be excluded from RNA editing. These results demonstrate that appropriately analyzed mRNA-seq data can recover a large proportion of high-confidence plastid RNA editing sites with high accuracy.

**Figure 5:**
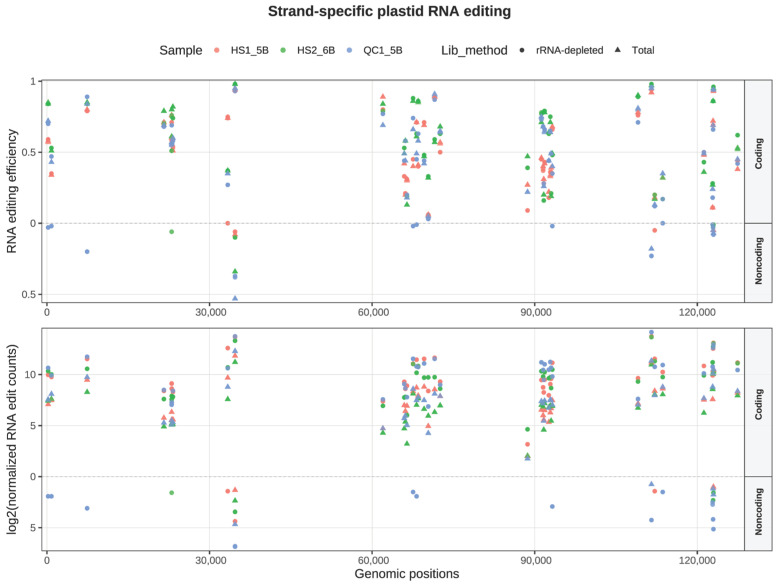
Strand-specific plastid RNA editing across the 38 high-confidence sites. (**Top**) editing efficiency; (**Bottom**) log2-normalized counts of edited reads. Points are colored by sample and shaped by library type (circles, rRNA-depleted RNA-seq; triangles, Total RNA-seq). Signals on the coding strand are plotted above the x-axis by genomic position; mirrored noncoding-strand signals are shown below.

Since both strands of plastid DNA are transcriptionally active, we further investigated whether RNA editing also occurs on transcripts complementary to coding genes. Among the 38 high-confidence sites, 15 exhibited nucleotide substitutions on both coding and noncoding strands. However, unlike the coding-strand variants, which were consistently detected across all six experiments, noncoding-strand substitutions were only observed in a few datasets (Figure 5). Moreover, their editing frequencies were substantially lower than those on the coding strand at the same positions, typically ranging from approximately 1 in 2840 to 1 in 63 reads. Notably, all noncoding-strand substitutions were G→A, which are complementary to the C→U changes on the coding strand. This pattern suggests that the apparent noncoding-strand signals likely arose from incomplete degradation of the second cDNA strand during the dUTP-based library preparation. Collectively, these findings reinforce the conclusion that bona fide plastid RNA editing is strand-specific and occurs exclusively on the coding strand.

## 4. Discussion

### 4.1. Divergent RNA Editing Landscapes Between G. uralensis and Vigna radiata

In this study, we report the first systematic identification of plastid RNA editing in the medicinal plant *G. uralensis*, establishing a high-confidence set of 38 editing sites. A comparative analysis with *V. radiata*, which possesses 41 known editing sites [14], revealed both conserved and lineage-specific characteristics. Although the total number of sites is similar between the two species, their distribution differs markedly. Specifically, *G. uralensis* undergoes editing in *ndhF*, *ndhG*, *ndhH*, *psbL*, *psbZ*, and *ycf2*, but is unedited in *V. radiata*. Conversely, editing occurs uniquely in *V. radiata* at *clpP*, *rps2*, *rps18*, and *rpl23*. Furthermore, *V. radiata* exhibits a greater number of edited codons in *ndhB* (10 versus 8 in *G. uralensis*). Divergent editing patterns were also observed at the *ndhD* locus: in *G. uralensis*, editing affects UCG, CCA, and UCA codons, whereas in *V. radiata*, the edited codons include ACA, ACG, and two distinct UCA sites.

The plastid ndh genes encode subunits of the thylakoid NDH complex, which supports cyclic electron flow around PSI and chlororespiration [28,29]. Under fluctuating or stressful environmental conditions, this complex contributes to photoprotection and redox homeostasis. RNA editing in *ndh* transcripts can modify amino acids, modulate protein function and is associated with environmental adaptation across plant lineages [30,31,32]. In *G. uralensis*, RNA editing events observed in multiple *ndh* genes—including *ndhF*, *ndhG*, *ndhH*, *ndhB*, and *ndhD*—may reflect adaptation to drought and heat stress typical of its native habitat, suggesting potential adaptive significance.

Such interspecific variation in plastid RNA editing has been widely reported across angiosperms [6,33,34], including within Fabaceae [14,15]. These differences may stem from lineage-specific acquisition or loss of editing sites [9,19,35] or changes in cis-elements accompanied by turnover of trans-acting factors such as PPR proteins [35,36]. Whether the divergence observed here reflects neutral evolutionary processes or adaptive responses to environmental pressures remains unclear. Notably, *G. uralensis* inhabits arid and often saline–alkali soils, conditions under which RNA editing in plastid genes, particularly those related to the NDH complex [37,38,39], may contribute to the modulation of redox homeostasis and stress tolerance. Further studies linking editing efficiency at specific sites to environmental factors, along with expression profiling of nuclear-encoded editing factors, could help clarify the roles of genetic divergence and adaptive plasticity in shaping the RNA editing landscape.

### 4.2. Distinguishing Authentic RNA Editing from Multiple Sources of Technical Artifacts

Accurate identification of bona fide plastid RNA editing events requires rigorous filtering of technical artifacts. Potential sources of false positives include organellar genomic polymorphisms, RNA base modifications, residual second-strand cDNA incorporation in dUTP-based libraries, and mapper- or caller-specific biases. In this study, we assembled the plastid genome for each sample, mitigating the complications of genomic polymorphism. Nevertheless, through systematic analysis, we identified and excluded four major classes of artifacts.

First, the misalignment of reads [40] spanning intron–exon junctions can generate spurious mismatches that resemble RNA editing sites near splice boundaries. We eliminated four such artifacts by cross-referencing gene annotations and filtering mismatches adjacent to splice junctions. Second, incomplete removal of the second cDNA strand in dUTP-based libraries [11,41] can lead to antisense signals complementary to true coding-strand editing events, manifesting as G→A changes on the noncoding strand (Figure 5). These artifacts were characterized by inconsistent detection across replicates, low variant fractions, limited read depth, and strand complementarity. They were filtered by enforcing strand specificity and minimum read-support thresholds. Third, we identified 48 variant sites within rRNA genes, predominantly on the noncoding strand and involving noncanonical substitutions. These likely arise from reverse transcriptase misincorporation opposite modified rRNA bases [42,43,44,45,46,47], with residual second-strand signals contributing to the detection of noncoding strands in rRNA-depleted libraries. Although such sites typically fall below detection thresholds in total RNA due to proportions (<1%) of rRNA with base modifications, we conservatively excluded all rRNA-mapping, noncanonical [48], and noncoding-strand-dominant calls as technical artifacts. Fourth, in rare instances, REDItools misreported C→U changes as G→A. We corrected six such sites (three in *ndhD*, one in *ndhF*, one in *ndhH* and one in intergenic) via manual inspection of strand-specific read counts across replicates.

Together, these filtering steps underscore how true plastid RNA editing events are confined to the coding strand, consisting primarily of C→U and infrequent U→C changes, and are supported by sufficient read depth and reproducibility. Although rRNA-derived variants were excluded as technical artifacts in the analysis of RNA editing, we acknowledge that dynamic rRNA modifications may contribute to ribosomal heterogeneity and cellular adaptation [49,50]: a biologically significant phenomenon distinct from canonical plastid RNA editing.

### 4.3. Evaluating Library Strategies: Superior Performance of Total RNA-Seq and Utility of mRNA-Seq in RNA Editing Identification

Although rRNA-depleted RNA-seq is often considered advantageous for organellar transcriptome studies, our findings demonstrate that total RNA-seq offers comparable sensitivity, higher reproducibility, and significantly fewer rRNA-derived artifacts for detecting plastid RNA editing.

Total RNA-seq detected 37 out of 38 high-confidence editing sites (editing efficiency >10%) consistently across all three replicates, whereas rRNA-depleted RNA-seq recovered 35 editing sites. Artifactual rRNA-associated variants were minimal in total RNA-seq (only 3 sites in total, with 2 reproducible across all samples), and substantially more prevalent in rRNA-depleted libraries (47 sites, only 2 of which were reproducible in all samples). In both library types, authentic editing events were consistently confined to the coding strand. In contrast, most rRNA-linked variants in rRNA-depleted libraries exhibited noncoding-strand dominance (46 out of 47), which is consistent with technical artifacts derived from rRNA modifications and library construction.

These results collectively indicate that total RNA-seq is a more robust strategy for plastid RNA-editing studies. It better preserves native transcriptomic architecture—including strand specificity and abundance ratios—thereby improving the accuracy of editing quantification and enhancing the biological interpretability of results.

Additionally, although standard poly(A)-selected mRNA-seq is inherently suboptimal for capturing non-polyadenylated plastid transcripts, our results demonstrate that even without organellar enrichment, this method can still reliably recover a substantial subset of editing sites. Specifically, 26 out of 38 high-confidence sites were consistently detected across libraries, and 34 sites were identified in the union of any two replicates despite lower overall mapping efficiency. This reliable detection might be attributed to the polycistronic nature of plastid transcription and the transient polyadenylation of plastid RNAs during degradation [15,51,52], which enables partial capture by poly(A)-based protocols. These findings suggest that, when combined with stringent strand-aware bioinformatic filtering, poly(A)-selected mRNA-seq can yield a credible plastid RNA editing strategy. Nevertheless, for comprehensive and artifact-resistant genome-wide profiling, total RNA-seq remains the superior approach. Importantly, the vast amount of publicly available mRNA-seq data represents a valuable and largely untapped resource that could be repurposed for large-scale exploratory analyses of RNA editing.

## Figures and Tables

**Table 1:** Statistics of whole-genome resequencing data and plastid assembly for the three *G. uralensis* samples.

Sample	Raw Reads	Plastid Genome	Plastid-Mapped Reads	Plastid Mapping Ratio
HS1_5B	292,305,370	127,702 bp	16,070,696	5.49%
HS2_6B	252,953,068	127,716 bp	7,911,608	3.12%
QC1_5B	327,037,602	127,670 bp	19,483,022	5.95%

**Table 2:** Statistics of RNA-seq data and mapping to the assembled plastid genomes for the three *G. uralensis* samples.

Sample	Library	Raw Reads	Plastid-Mapped Reads	Plastid Mapping Ratio
HS1_5B	Total RNA-seq	127,089,854	32,020,931	25.19%
HS1_5B	rRNA-depleted RNA-seq	70,544,244	23,316,279	33.05%
HS1_5B	mRNA-seq	100,743,972	434,781	0.43%
HS2_6B	Total RNA-seq	123,558,206	25,053,748	20.27%
HS2_6B	rRNA-depleted RNA-seq	63,621,904	14,703,453	23.11%
HS2_6B	mRNA-seq	103,140,076	147,072	0.14%
QC1_5B	Total RNA-seq	112,646,262	33,606,579	29.83%
QC1_5B	rRNA-depleted RNA-seq	66,603,282	26,073,981	39.14%
QC1_5B	mRNA-seq	124,564,614	608,462	0.48%

**Table 3:** Summary of the 34 plastid RNA editing sites in coding sequences of *G. uralensis* by gene.

Gene	Number of Editing Sites
*accD*	2
*atpA*	1
*ndhA*	2
*ndhB*	8
*ndhD*	3
*ndhE*	1
*ndhF*	1
*ndhG*	2
*ndhH*	1
*petB*	2
*petL*	1
*psaI*	1
*psbF*	1
*psbL*	1
*psbZ*	1
*rpoA*	1
*rpoB*	4
*rps14*	1

Note: This table only includes sites within coding sequences (CDSs), totaling 34 sites. Four additional sites—one site in an *ndhA* intron, one in the *psbF* 5′ UTR, one in the *psbT* 3′ UTR, and one intergenic region—were identified but are excluded here for consistency with Table 4.

**Table 4:** Summary of the 34 RNA-editing codons in the plastid genome of *G. uralensis*.

Gene	Position on Gene	Codon Change	Amino Acid Change	Codon Position	Editing Efficiency	Supporting Reads
*accD*	794	TCG→TTG	S→L	2	0.34~0.53	362~1036
*accD*	1403	CCT→CTT	P→L	2	0.57~0.84	273~1684
*atpA*	791	CCC→CTC	P→L	2	0.79~0.89	611~3605
*ndhA*	341	TCA→TTA	S→L	2	0.57~0.9	156~3556
*ndhA*	1073	TCT→TTT	S→F	2	0.42~0.71	193~3303
*ndhB*	95	TCA→TTA	S→L	2	0.45~0.77	188~2474
*ndhB*	413	CCA→CTA	P→L	2	0.38~0.78	129~1469
*ndhB*	532	CAT→TAT	H→Y	1	0.16~0.31	47~751
*ndhB*	692	TCT→TTT	S→F	2	0.42~0.79	182~2172
*ndhB*	782	TCA→TTA	S→L	2	0.18~0.64	81~1491
*ndhB*	1058	TCA→TTA	S→L	2	0.34~0.75	157~2515
*ndhB*	1201	CAT→TAT	H→Y	1	0.19~0.49	87~1711
*ndhB*	1427	CCA→CTA	P→L	2	0.35~0.68	234~2531
*ndhD*	383	CCA→CTA	P→L	2	0.13~0.31	18~538
*ndhD*	674	TCG→TTG	S→L	2	0.2~0.58	81~438
*ndhD*	878	TCA→TTA	S→L	2	0.33~0.53	51~696
*ndhE*	230	CCG→CTG	P→L	2	0.4~0.88	546~3135
*ndhF*	290	TCA→TTA	S→L	2	0.69~0.89	38~199
*ndhG*	50	TCG→TTG	S→L	2	0.4~0.86	393~1980
*ndhG*	347	CCA→CTA	P→L	2	0.45~0.71	253~3126
*ndhH*	505	CAT→TAT	H→Y	1	0.5~0.68	246~702
*petB*	12	GTC→GTT	V→V	3	0.12~0.2	446~3317
*petB*	611	CCA→CTA	P→L	2	0.92~0.98	3888~19,280
*petL*	5	CCG→CTG	P→L	2	0.36~0.5	147~2038
*psaI*	79	CAT→TAT	H→Y	1	0.38~0.62	484~2556
*psbF*	77	TCT→TTT	S→F	2	0.66~0.86	1960~6852
*psbL*	2	ACG→ATG	T→M	2	0.93~0.96	2429~9780
*psbZ*	50	TCC→TTC	S→F	2	0.27~0.74	376~6873
*rpoA*	200	TCT→TTT	S→F	2	0.71~0.9	206~889
*rpoB*	338	TCT→TTT	S→F	2	0.51~0.82	68~361
*rpoB*	551	TCA→TTA	S→L	2	0.55~0.8	62~442
*rpoB*	566	TCG→TTG	S→L	2	0.51~0.76	66~620
*rpoB*	2000	TCT→TTT	S→F	2	0.68~0.79	59~382
*rps14*	80	CCA→CTA	P→L	2	0.93~0.98	4660~14,663

## Data Availability

The data that support the findings of this study have been deposited into CNSA [1] with accession number CNP0007853.
